# Close of the Year

**Published:** 1880-12

**Authors:** 


### Editorial.

### CLOSE OF THE YEAR.

With this number we close the calendar year, but not this volume of the JOURNAL. County, State and National Medical Associations have gone through their annual rounds, and much of their transactions is found on our pages. Colleges have arrived at the holidays of their course, and the tired student is anxious to leave the lecture room for a few days of recreation and pleasure with friends and relatives at home.

So far as our information extends the attendance in various Institutions is measurably full. The close in March will, therefore, furnish the usual number of authorized recruits to the medical army. We look back upon the year's labor without regret for any prominent or fatal error it is true, but with a determination to make ourselves, if possible, more useful to mankind during the year 1881.

Age sobers down zeal and ardor, but whets the sense of responsibilities and the duties we owe to others. We have endeavored during the year to mention, editorially, each month, that which seemed of most importance to the profession. The November number alluded to the relations between druggists and physicians, which should be dispassionately considered, and a decision made, forever settling the wrangle. During the year other important subjects have been presented, amongst them the probable benefit that may be derived from the local application o carbolic acid to cancerous ulcerations.

To all of these subjects we again invite special attention.

